# Characterization of *Mycobacterium orygis*

**DOI:** 10.3201/eid1810.120569

**Published:** 2012-10

**Authors:** Nicolaas C. Gey van Pittius, Paul D. van Helden, Robin M. Warren

**Affiliations:** Stellenbosch University Faculty of Medicine and Health Sciences, Tygerberg, South Africa

**Keywords:** Mycobacterium tuberculosis complex, Mycobacterium orygis, oryx bacillus, genotyping, characterization, bacteria, tuberculosis and other mycobacteria

**To the Editor:** In a recently published study, van Ingen et al. (*1*) described the molecular characterization and phylogenetic position of the oryx bacillus, a member of the *Mycobacterium tuberculosis* complex, and proposed a long overdue name for the organism: *Mycobacterium orygis*. The authors described oryx bacillus as a separate taxon; the aim was for this description to be used in the future to identify the subspecies. Thus, we thought it pertinent to provide additional information that would be useful in speciating isolates of the oryx bacillus.

In a recent study, we genotyped an isolate of oryx bacillus obtained from an African buffalo in South Africa (*2*). This isolate was typed by using 16S rDNA, *M. tuberculosis* complex–specific multiplex-PCR, regions-of-difference analyses, *gyrase B* gene single nucleotide polymorphism (SNP) analysis, spoligotyping, and mycobacterial interspersed repetitive units–variable number tandem repeat typing. We showed that, in addition to the markers described by van Ingen et al. (*1*), regions of difference 701 and 702 were also intact in *M. orygis*.

In addition, van Ingen et al. identified the *Rv2042^38^* GGC mutation as a novel, useful genetic marker to identify *M. orygis*. However, such a marker already exists in the form of the very specific *gyrB^oryx^* G to A SNP at position 1113, which was described by Huard et al. (*3*). On its own, SNP detection in the *gyrB* gene allows differentiation of at least 6 of the 9 *M. tuberculosis* complex species from each other (*M. canettii*, *M. tuberculosis*, *M. orygis*, *M. microti*, *M. caprae*, and *M. bovis*) (*3*). Thus, the SNP at position 1113 is more useful than the Rv2042^38^ mutation as a novel and distinct genetic marker to identify *M. orygis*.

Apart from this, we found that the sequence type (ST) 587 was not the only spoligotype specific for *M. orygis*. In our study, the variant type ST701 (annotated as *M. africanum* in the spolDB4 database) (*4*) is also an *M. orygis*–specific type and exactly matches that of a previous isolate of the oryx bacillus (SB0319) from the *M. bovis* spoligotype database (*5*). This spoligotype differs from ST587 by the presence of spacer 18, and the spoligotype was not found in the extensive sample set of van Ingen et al. (*1*).
